# Controlled Prospective Evidence of Rapid Maxillary Expansion Efficacy in Pediatric Obstructive Sleep Apnea: A Systematic Review Update

**DOI:** 10.3390/jcm15082976

**Published:** 2026-04-14

**Authors:** Marcos Fernández-Barriales, Montserrat López de Luzuriaga, Irene Lafuente-Ibáñez de Mendoza, Juan Julián Alonso Fernández-Pacheco, Ainhoa Álvarez Ruiz de Larrinaga, José Manuel Aguirre Urizar

**Affiliations:** 1 Bioaraba, Grupo de Investigación en Apneas y Trastornos del Sueño, 01009 Vitoria-Gasteiz, Spain; ainoa.alvarezruizdelarrinaga@osakidetza.eus; 2 Osakidetza, Hospital Universitario Araba, Servicio de Cirugía Oral y Maxilofacial, 01009 Vitoria-Gasteiz, Spain; juanjulian.alonsofernandez-pacheco@osakidetza.eus; 3 Departamento de Cirugía, Medicina Física y Radiología, Facultad de Medicina, Universidad del País Vasco (UPV-EHU), 01009 Vitoria-Gasteiz, Spain; 4 Osakidetza, Hospital Universitario Araba, Unidad Funcional del Sueño, 01009 Vitoria-Gasteiz, Spain; 5 Departamento de Estomatología, Universidad del País Vasco (UPV/EHU), 48940 Leioa, Spain; montserratsofia.lopezdeluzuriaga@ehu.eus (M.L.d.L.); irene.lafuente@ehu.eus (I.L.-I.d.M.); josemanuel.aguirre@ehu.eus (J.M.A.U.)

**Keywords:** palatal expansion technique (MESH), rapid maxillary expansion, obstructive, sleep apnea (MESH), natural history, children

## Abstract

**Background/Objectives**: In our previous systematic review, we found no convincing evidence to support rapid maxillary expansion (RME) as a treatment for pediatric obstructive sleep apnea (OSA). Subsequent clinical guidelines and expert opinion have continued to recommend this orthodontic treatment. We aimed to update our search to determine whether new controlled prospective evidence alters this conclusion. **Methods**: We updated our previous systematic review, searching for controlled prospective studies on RME efficacy for pediatric OSA published up to 1st February 2026. **Results**: Three new randomized clinical trials (RCTs) were identified in addition to the five original studies. Critically, none of the new RCTs included a watchful waiting or supportive care control arm, leaving the sole available comparison against watchful waiting unchanged: a single RCT that found no significant difference in AHI change between RME and observation. Of the remaining new RCTs, two found marginal AHI improvement with RME and one found no significant AHI reduction. **Conclusions**: The observed AHI improvements in studies lacking an untreated comparator cannot be distinguished from the known potential for spontaneous improvement in growing children. Extreme caution is warranted before recommending RME as a treatment for pediatric OSA.

## 1. Introduction

Obstructive sleep apnea (OSA) represents the most severe manifestation of sleep-related disordered breathing (SRDB) [1] and affects 1–4% of the pediatric population [2]. Polysomnography is considered the gold standard for diagnosing pediatric OSA, with the apnea–hypopnea index (AHI) being the most widely used measure for both diagnosis and evaluating treatment outcomes [3]. Adenotonsillectomy (T&A)—the current gold standard treatment [4,5]—fails to improve AHI in over 20% of the patients [6]. Furthermore, a considerable proportion of patients may spontaneously improve [7,8,9,10], sparing a surgical intervention not free of morbidity [11]. Less invasive effective treatment alternatives are needed [12].

Rapid maxillary expansion (RME), a non-invasive orthodontic intervention aimed at correcting maxillary constriction, is often reported as an alternative therapeutic approach for managing pediatric OSA. In our previous systematic review, we found no compelling evidence to substantiate the efficacy of RME compared to other treatment alternatives or even watchful waiting [12]. However, subsequent expert opinion and clinical guidelines continue to recommend RME as a suitable treatment alternative for this disease [13,14,15,16].

We therefore sought to answer the following question: in children (<18 years old) with OSA, does RME improve sleep study outcomes compared to watchful waiting or alternative treatment?

## 2. Materials and Methods

### 2.1. Protocol and Registration

The protocol for this systematic review was first registered in PROSPERO (CRD42021249261) on 18 June 2021, and it was later updated on 15 October 2025. We followed the PRISMA 2020 guidelines [17] (see Supplementary Table S1).

### 2.2. Eligibility Criteria

Studies were included if they were experimental, such as randomized clinical trials, or quasi-experimental, like prospective longitudinal non-randomized controlled studies. The exclusion criteria were: (1) studies with adult patients, surgically assisted techniques or concomitant treatment in the intervention group, no diagnosis of OSA, no sleep study (PSG or HSAT), no data on the primary outcome (pre- and post-treatment AHI); (2) studies with patients with craniofacial, cardiorespiratory or neurological syndromes, or healthy non-OSA control patients; (3) case reports, reviews, opinions, etc.; and (4) in vitro and in vivo studies. The PICO question was: children diagnosed with OSA by PSG or HSAT (population); orthodontic maxillary expansion by means of an intraoral device intervention); watchful waiting or alternative treatment (comparison); and change between pre- and post-treatment AHI as measured in a sleep study (PSG or HSAT) (outcome).

### 2.3. Information Sources and Search Strategy

A search for literature was conducted electronically using PubMed, Web of Science, Embase, and Cochrane Central databases. References from original papers and review articles were cross-checked to identify additional trials. No limitation on language was considered. Authors were contacted if data was missing or incomplete. The search was updated for articles published from 1 December 2021 until 1 February 2026 (Supplementary Table S2).

### 2.4. Selection Process

Two independent reviewers (M.F.-B. and I.L.-I.d.M.) systematically and independently assessed both the titles and abstracts of all identified records for inclusion and exclusion criteria. If an abstract failed to provide sufficient information to reach a decision, the full text was retrieved. If reviewers disagreed, a third party (J.M.A.U.) was brought in to resolve the issue.

### 2.5. Data Collection Process and Items

The extraction of qualitative and quantitative data was performed using a structured data extraction form (Excel datasheet; Microsoft Corporation, Redmond, WA, USA, version 2021) by one investigator (M.F.-B.), and it was double-checked by another (I.L.-I.d.M.) (Supplementary Table S3). The data from pre- (T_0_) and post-treatment (T_1_) sleep study parameters in intervention (RME) and control groups were extracted: AHI (main outcome); lowest oxygen saturation, mean oxygen saturation (secondary outcomes). Researchers also collected data on the percentage change in AHI before and after the treatment, the proportion of patients with residual disease following the treatment, and the time elapsed between the initial and final sleep studies. If the data on AHI change was not explicitly available, it was manually calculated as the percentage of the difference between pre- and post-treatment AHI means = (AHI T_0_ mean − AHI T_1_ mean)/AHI T_0_ mean × 100 or AHI medians = (AHI T_0_ median − AHI T_1_ median)/AHI T_0_ median × 100. Cure rate, defined as an AHI < 1, was also manually calculated if unavailable as the rate of (patients with AHI T_1_ < 1)/(total of patients at T_1_) × 100. Readers should note that group-level calculations do not capture individual variability and may introduce imprecision in effect estimates.

### 2.6. Risk of Bias

Two reviewers (M.F.-B. and I.L.-I.d.M.) independently assessed the risk of bias using the modified Joanna Briggs Institute (JBI) critical appraisal checklist for randomized controlled trials and case–control studies [18]. In case of disagreement, a third reviewer was consulted (J.M.A.U.).

### 2.7. Certainty of Evidence Assessment

The GRADE framework (Grading of Recommendations Assessment, Development, and Evaluation) [19] was used to assess the certainty of the evidence across the domains of risk of bias, inconsistency, indirectness, imprecision, and publication bias. Randomized controlled trials were originally considered to provide high-certainty evidence, while observational studies were regarded as low-certainty.

## 3. Results

### 3.1. Study Selection

**Figure 1:**
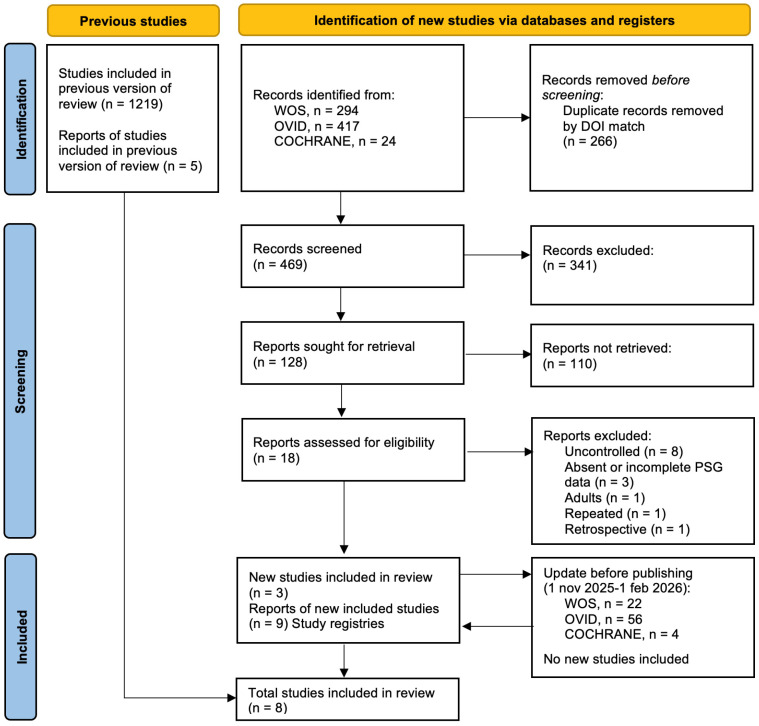
PRISMA 2020 flow diagram for updated systematic reviews.

The updated search yielded 735 new records, 469 unduplicated. A total of 451 articles were eliminated: 341 during the title screening and 110 during the abstract screening, leaving 18 articles for a full-text review. At the end, three new studies [20,21,22] were added to the five studies [23,24,25,26,27] identified in the original systematic review [12]. A qualitative analysis was conducted. Notably, none of the three new studies included a watchful waiting or supportive care control arm—a critical gap that leaves the fundamental question of RME efficacy over natural history unanswered. The selection process is outlined in Figure 1, and rejected articles, along with their reasons for exclusion, are provided in Supplementary Table S4.

### 3.2. Study Characteristics

Methodology of the studies was heterogeneous and included three parallel RCTs [20,22,24], two cross-over RCTs [21,23] and three non-randomized controlled longitudinal cohorts [25,26,27] (see Table 1).

**Table 1:** General characteristics of included studies.

Authors [Reference]	Country	Study Design	Sleep Study	Control Group	RME (*n*)	Follow-Up (Months)
Guilleminault et al. [23]	France, Italy	Cross-over RCT	PSG	T&A	31	RME: 3 T&A: 1
Hoxha et al. [24]	Turkey	Parallel RCT	HSAT	WW	15	RME: 5.01 (0.96) WW: 5.38 (1.36)
Pirelli et al. [25]	Italy	NRC	HSAT	T&A	40	4
Villa et al. 2014 [26]	Italy	NRC	PSG	T&A	22	12
Villa et al. 2016 [27]	Italy	NRC	PSG	T&A MT	21	>6
Gokce et al. [20]	Turkey	Parallel RCT	HSAT	3 devices	46	3
Magalhaes et al. [21]	Brazil	Crossover RCT	PSG	T&A RME	32	1st check: 6 2nd check: 12
Aksilp et al. [22]	Thailand	Parallel RCT	PSG	T&A	12	6

RME: rapid maxillary expansion; RCT: randomized controlled trial; HSAT: home sleep apnea test; PSG: polysomnography; T&A: adenotonsillectomy. NRC: non-randomized controlled; WW: watchful waiting; MT: Medical Treatment. Grey shading indicates studies included in the original review [12].

### 3.3. Results of Individual Studies

#### 3.3.1. Demography and Risk Factors

We analyzed 366 patients, 219 of whom underwent RME. The studies included patients between 6 [22] and 13 [20] years old. Two studies [22,26] reported a higher number of male patients, while another study [20] found more female patients. Two studies did not report the BMI data [23,24]. Gokce et al. presented patients in the upper range of normality for Turkish 12–13-year-olds [20]. Race, socioeconomic status, or pre-term birth was not reported. One patient dropped out before treatment in the crossover RCT conducted by Guilleminault et al. [23], and another patient was lost to follow-up in the crossover RCT conducted by Magalhaes et al. due to an excessive delay before second line therapy [21]. The pediatric OSA diagnosis was confirmed through full-night PSG [21,22,23,26,27] or home sleep apnea test (HSAT) [20,24,25]. See Table 2.

**Table 2:** Anthropometric and demographic characteristics of included studies.

Authors [Reference]		Gender (M/F)	Age Mean (SD), Years	BMI Mean (SD), kg/m^2^
Guilleminault et al. [23]		14/17	6.5 (0.2) *	-
Hoxha et al. [24]	RME WW	14/16	12.27 (1.93) 11.46 (2.06)	-
Pirelli et al. [25]		43/37	7.1 (0.8) *	<24
Villa et al. 2014 [26]	RME T&A	34/13	6.58 (1.83) ^†^ 3.7 (0.92) ^†^	18.82 (3.44) ^†^ 15.75 (1.82) ^†^
Villa et al. 2016 [27]	RME T&A MT	44/32	6.16 (1.68) 4.54 (1.69) 4.34 (1.08)	19.91 (2.23) 16.98 (3.29) 15.86 (1.53)
Gokce et al. [20]	TTB TB BB	16/30	12.5 12.8 13.1	22.2 22.8 23.2
Magalhaes et al. [21]	T&A RME	15/17	8.9 (2.3) 8.1 (1.1)	16.08 (0.62) 15.77 (0.86)
Aksilp et al. [22]	T&A	15/9	6.33(1.71)	16.22(2.8)

M: Male; F: Female; SD: Standard deviation; BMI: Body mass index; -: Not reported; RME: Rapid maxillary expansion; WW: Watchful waiting; MT: Medical Treatment; TTB: tooth-tissue-borne; TB: tooth-borne; BB: Bone-borne; T&A: Adenotonsillectomy. * Mean age of full sample provided only. ^†^ Statistically significant difference between T&A and RME cohorts. Grey shading indicates studies included in the original review [12].

#### 3.3.2. Intervention and Controls

All studies performed RME with a fixed endo-oral appliance: Gokce et al. compared tooth, tooth–tissue, and bone anchorage [20], while the rest described standard tooth anchorage [21,27]. Supplementary Table S5 provides device details and activation regimes.

Aksilp et al. reported no adverse effects or complications in either RME or adenotonsillectomy treatment arms [22]. Gokce et al. reported mild gingivitis in relation to plaque aggregation [20].

#### 3.3.3. Outcomes

Both crossover RCTs [21,23] were divided into two temporal groups: an initial cohort evaluating primary RME versus adenotonsillectomy and a subsequent cohort comparing secondary adenotonsillectomy after RME versus secondary RME after adenotonsillectomy. The main outcome, AHI, was extracted before (T_0_) and after (T_1_) treatment. Table 3 provides AHI values before (T_0_) and after (T_1_) treatment, AHI change percentage, and cure rate. Secondary outcomes (LSAT and MSAT) are reported in Table 4.

**Table 3:** Sleep study primary outcomes: AHI pre- and post-intervention, percentage cured, and AHI change percentage.

Authors [Reference]	Treatment Arm	AHI at T_0_ Mean (SD)	*p*	AHI at T_1_ Mean (SD)	% Cured T_1_ AHI < 1	% ΔAHI *
Guilleminault et al. [23] First line	RME	11.1 (0.7)	0.00	5.4 (0.6)	7% (1/15)	51% *
	*p*	0.2	0.53	0.15	-	-
	T&A	12.5 (0.8)	0.00	4.9 (0.6)	0% (0/16)	60% *
Guilleminault et al. [23] Second line	RME after T&A	4.9 (0.6)	0.00	0.9 (0.3)	93% (13/14)	81% *
	*p*	0.15	0.49	0.16	-	-
	T&A after RME	5.4 (0.6)	0.00	0.9 (0.3)	94% (15/16)	83% *
Hoxha et al. [24]	RME	2.5 (1.12)	<0.05	1.79 (1.05)	-	28% *
	*p*	-	n.s.	-	-	-
	WW	2.67 (1.23)	<0.05	1.8 (1.08)	-	33% *
Pirelli et al. [25]	RME	12.1 (4.9)	-	5.4 (5.4)	37% (15/40)	55%
	*p*	-	-	-	-	-
	T&A	-	-	-	15% (6/40)	-
Villa et al. 2014 [26]	RME	5.81 (6.05)	0.005	2.64 (3.11)	37% (8/22)	36% (74.63)
	*p*	0.000	-	0.468	0.408	0.011
	T&A	17.25 (13.94)	0.000	1.79 (1.82)	44% (11/25)	84% (17.79)
Villa et al. 2016 [27] (RME vs. T&A)	RME	5.6 (1.8–17.2)	<0.005	1.9 (0–11.8)	38% (8/21)	66% *
	*p*	-	-	-	-	-
	T&A	16.3 (6–71.5)	<0.005	1.3 (0–11.9)	31% (13/42)	92% *
Villa et al. 2016 [27] (RME vs. MT)	RME	5.6 (1.8–17.2)	<0.005	1.9 (0–11.8)	38% (8/21)	66% *
	*p*	-	-	-	-	-
	MT	4.4 (0.8–31.3)	n.s.	2.4 (0.6–21.1)	15% (2/13)	45% *
Gokce et al. [20]	TTB	9.2 (6.5–12.3) ^†^	0.280	8.0 (4.9–9.0)	-	17.39% *
	*p*	0.277		0.857	-	-
	TB	5.4 (1.5–12.6) ^†^	0.691	5.5 (2.7–15.1)	-	−11.11% *
	*p*	0.277		0.857	-	-
	BB	5.8 (3.6–10.7) ^†^	0.796	5.1 (4.2–8.7)	-	8.62% *
Magalhaes et al. [21] First line	T&A first	8.1 (6.6)	-	2.21	-	72.71% *
	*p*	0.075	-	-		
	RME first	12.5 (8.0)	-	8.36	-	33.12% *
Magalhaes et al. [21] Second line	RME after T&A	2.21	-	2.13	-	3.62% *
	*p*	-	-	-	-	-
	T&A after RME	8.36	-	4.05	-	51.55% *
Aksilp et al. [22]	RME	6.8 [5.6–8.1] ^‡^	<0.01	2.3 [1.15–5.7] ^‡^	16.7%	66.2% *
	*p*	>0.05	-	0.07	0.37	-
	T&A	7 [5.25–9.9] ^‡^	<0.01	1.4 [0.7–1.85] ^‡^	41.7%	80% *

AHI: apnea–hypopnea index; SD: standard deviation; % ΔAHI: percentage of change in AHI between T_0_ and T_1_ assessments; -: not reported; WW: watchful waiting; TTB: tooth tissue-borne; TB: tooth-borne; BB: bone-borne; T&A: adenotonsillectomy; RME: rapid maxillary expansion; MT: medical treatment; n.s.: not significant. * % ΔAHI not reported, calculated as the percentage of the difference in pre- and post-treatment AHI means= (AHI T_0_ mean − AHI T_1_ mean)/AHI T_0_ mean × 100, or medians = (AHI T_0_ median − AHI T_1_ median)/AHI T_0_ median × 100. ^†^ mean (95% confidence interval). ^‡^ median [interquartile range]. Grey shading indicates studies included in the original review [12].

**Table 4:** Sleep study secondary outcomes: mean oxygen saturation (MSAT), lowest oxygen saturation (LSAT).

Authors [Reference]	Treatment Arm	MSAT at T_0_ Mean (SD)	*p*	MSAT at T_1_ Mean (SD)	LSAT at T_0_ Mean (SD)	*p*	LSAT at T_1_ Mean (SD)
Guilleminault et al. [23] First line	RME	-	-	-	92.5 (0.4)	0.00	95.9 (0.3)
	*p*	-	-	-	0.53	0.15	0.65
	T&A	-	-	-	92.1 (0.5)	0.00	95.2 (0.3)
Guilleminault et al. [23] Second line	RME after T&A	-	-	-	95.2 (0.3)	0.00	98 (0.2)
	*p*	-	-	-	0.65	0.68	0.004
	T&A after RME	-	-	-	95.9 (0.3)	0.00	97.6 (0.3)
Hoxha et al. [24]	RME	96.31 (0.75)	n.s.	96.08 (0.64)	88.08 (4.32)	n.s.	89.75
	*p*	-	-	-	-	n.s.	-
	WW	95.87 (1.36)	n.s.	95.8 (1.15)	89 (4.49)	n.s.	89.5 (3.63)
Pirelli et al. [25]	RME	-	-	-	84.6 (2.7)	-	95.2 (3.5)
	*p*	-	-	-	-	-	-
	T&A	-	-	-	-	-	-
Villa et al. 2014 [26]	RME	96.56 (0.47)	0.013	97.42 (1.84)	-	-	-
	*p*	n.s.	-	n.s.	-	-	-
	T&A	96.11 (2.7)	0.013	97.5 (1.14)	-	-	-
Villa et al. 2016 [27] (RME vs. T&A)	RME	97.29 (1.49)	<0.005	97.62 (0.86)	-	-	-
	*p*	-	-	-	-	-	-
	T&A	96.47 (1.79)	<0.005	98.03 (0.79)	-	-	-
Villa et al. 2016 [27] (RME vs. MT)	RME	97.29 (1.49)	<0.005	97.62 (0.86)	-	-	-
	*p*	-	-	-	-	-	-
	MT	96.96 (1.16)	n.s.	97.37 (1.21)	-	-	-
Gokce et al. [20]	TTB	-	-	-	82 (78–87) ^†^	0.401	80 (70–85) ^†^
	*p*	-	-	-	0.713	-	0.557
	TB	-	-	-	81 (78–85) ^†^	0.887	84 (70–89) ^†^
	*p*	-	-	-	0.713	-	0.557
	BB	-	-	-	81 (66–86) ^†^	0.407	82 (75–88) ^†^
Magalhaes et al. [21] First line	T&A first	-	-	-	90.9 (2.7)	n.s.	92.5
	*p*	-	-	-	0.293		n.s.
	RME first	-	-	-	86.9 (9.6)	n.s.	88.6
Magalhaes et al. [21] Second line	RME after T&A	-	-	-	92.5	n.s.	91.7
	*p*	-	-	-	n.s.		n.s.
	T&A after RME	-	-	-	88.6	n.s.	91.2
Aksilp et al. [22]	RME	98 [97.5–98] ^‡^	0.37	98 [98–98] ^‡^	86 [83.5–90] ^‡^	0.12	89.5 [86–93] ^‡^
	*p*	>0.05	-	n.s.	>0.05	-	n.s.
	T&A	97.5 [96.5–98] ^‡^	0.75	98 [97–98] ^‡^	87 [81–87] ^‡^	<0.05	90.5 [89–92.5] ^‡^

MSAT: Mean oxygen saturation; LSAT: lowest oxygen saturation; SD: standard deviation; -: not reported; TTB: tooth tissue-borne; TB: tooth-borne; BB: bone-borne; T&A: adenotonsillectomy; n.s.: not significant; RME: rapid maxillary expansion; MT: medical treatment; WW: watchful waiting. ^†^ mean (95% confidence interval). ^‡^ median [interquartile range]. Shaded cells correspond to studies already identified in our original review [12].

#### 3.3.4. Dentofacial Inclusion Criteria

All studies reported maxillary transversal features as an indicator for the RME treatment: maxillary constriction [22,23,24,25,26,27], unilateral [21], or bilateral [20] posterior cross-bite (see Supplementary Table S5). Gokce et al. reported baseline upper intermolar average (95% confidence interval) widths in tooth-tissue-, tissue-, and bone-borne arms of 42.6 (40.99–44.21), 44.83 (42.56–47.10), and 42.69 (40.21–45.16) mm, that were widened to 47.65 (44.52–50.77), 47.98 (45.49–50.47), and 48.1 (44.98–51.21) mm, respectively, after treatment, corresponding to the average 5.04 (1.79–8.29), 3.15 (−1.10–7.41), and 5.41 (3.46–7.35) mm increase [20]. Magalhaes et al. reported an expansion goal of 2 to 3 mm overcorrection at the level of the upper molars but did not disclose either baseline or effectively attained maxillary transverse dimensions [21]. Aksilp et al. reported a baseline average intercanine width of 33.05 ± 2.06 mm in the RME arm, which increased to 37.18 ± 2.38 mm after 6 months [22].

#### 3.3.5. Quantitative Synthesis

A quantitative synthesis of the results was omitted because the included studies showed significant differences in methods and clinical characteristics. The variations in study designs, participant demographics, treatment comparators, and outcome measurement tools precluded a meaningful quantitative pooling of data. There were no new studies comparing the RME treatment with watchful waiting. Across all eight included studies, only one—the parallel RCT by Hoxha et al. [24]—directly compared RME with watchful waiting, and it found no statistically significant difference in AHI change between groups. This finding, combined with the absence of any watchful waiting or supportive care arm in the three new RCTs, constitutes the central finding of this review: the controlled prospective evidence does not demonstrate a meaningful advantage of RME over the natural course of the disease.

#### 3.3.6. Risk of Bias Assessment

The risk of bias was evaluated using the validated JBI critical appraisal tool [18] for randomized and quasi-randomized clinical trials [20,21,22,23,24,25,26,27], as shown in Supplementary Table S6.

#### 3.3.7. Certainty of Evidence Assessment

According to the GRADE assessment, the overall certainty of evidence was low to very low across all evaluated outcomes (see Supplementary Table S7) [20,21,22,23,24,25,26,27]. Downgrading was primarily due to risk of bias, small sample sizes, lack of adjustment for confounding in observational data, and imprecision of effect estimates.

## 4. Discussion

In our previous review of the literature, we concluded that the available controlled evidence was insufficient to associate the observed improvements in pediatric OSA patients with RME treatment [12]. The latest systematic review update shows that, despite new controlled studies being published, there is still no straightforward comparison between the effects of RME and simply letting children grow without intervention. As we thoroughly discussed in the original review, the fact that pediatric OSA has proven the ability for spontaneous improvement both in robust clinical trials [6] and prospective long-term population-based cohorts [7,8,9,10] renders control—preferably with watchful waiting or supportive care measures—paramount for claiming treatment efficacy [12]. The three new RCTs identified in this update do not resolve this uncertainty: none included a watchful waiting or supportive care arm, and their findings therefore cannot be interpreted as evidence of a genuine RME treatment effect over the natural course of the disease.

Beyond the absence of an appropriate comparator, the included studies exhibit substantial methodological heterogeneity. Sleep study modalities ranged from in-laboratory PSG to HSAT, which yield systematically different AHI values and are not directly comparable. Follow-up durations varied widely (3 to >12 months), which is particularly relevant, given the time-dependent nature of spontaneous OSA improvement in growing children. These sources of heterogeneity precluded any quantitative synthesis and must be considered when interpreting apparently discrepant results across studies.

We have found three new RCTs on this topic. Unfortunately, none of them specifically addressed the potential contribution of spontaneous improvement to the observed outcomes. The first RCT compared three different RME devices and found them equally ineffective in reducing AHI [20]. The other two compared RME to the gold standard treatment, adenotonsillectomy. Magalhaes et al. presented a crossover design, in which RME provided a marginal effect on the overall AHI improvement [21]. Aksilp et al. found that RME influence on AHI was comparable to that observed in patients that underwent adenotonsillectomy, although the latter provided a significantly higher symptomatic relief [22]. Unfortunately, none of these three RCTs allowed by design a proper assessment of the potential contribution of spontaneous improvement to the observed AHI changes. Therefore, the only study comparing RME to either a watchful waiting or supportive care approach identified with our full systematic review protocol to date was the randomized clinical trial by Hoxha et al. [24]. In this RCT, the authors did not find differences in their main outcome, the AHI change, between patients allocated to receive RME compared to those who followed their natural growth and development without an intervention. Taken together, these findings are internally consistent with our original conclusion [12]: where a proper control exists, RME shows no advantage over the comparator. Where no such control exists, the observed AHI improvements are indistinguishable from spontaneous improvement.

One study from our search update appears to address this critical need for a treatment-naïve control arm: Remy et al. compared a cohort of RME patients with an untreated age-matched cohort who “did not yet receive the orthopedic treatment” [28]. However, upon full text read and data extraction, the AHI and PSG data from both arms were only available at baseline, with longitudinal follow-up limited to the RME arm alone. Similarly, Alforaidi et al. presented a prospective study that seemed to compare RME treatment outcomes with an untreated cohort who received “occlusal interference relieves when needed” only. Although their main outcome was an indirect measure of urine inflammatory markers, they reported a reduction in AHI in the RME group of −3.53 ev.h^−1^ and a worsening of +1.15 ev.h^−1^ in the control group [29]. Such control group matched age, sex, race, body mass index, and cephalometric measurements but without explicit mention of the baseline AHI or PSG parameters. The absence of the baseline diagnostic criteria in this cohort warrants extreme caution when interpreting follow-up AHI comparisons or drawing conclusions about OSA treatment efficacy. Both studies illustrate a recurrent pattern in this literature: designs that superficially resemble controlled comparisons but lack the methodological rigor to isolate the effect of RME from baseline imbalance, spontaneous improvement, or natural growth—precisely the confounders that only a properly randomized arm can control.

According to our inclusion criteria, which required either an experimental or quasi-experimental design, retrospective cohorts were excluded. A retrospective study that featured a control arm with untreated patients gathered subjective sleep apnea symptoms with the PSQ questionnaire. Notably, the results were only available in the orthodontic treatment arm [30]. The absence of objective sleep study data and the restriction of symptom assessment to treated patients undermined the ability to draw robust conclusions regarding treatment efficacy for sleep apnea in the study cohort.

Another retrospective cohort presented a robust sample size (*n* = 1217) and looked at a clinically relevant population, persistent OSA after adenotonsillectomy: the authors compared the short- and long-term effects of RME and other popular treatment alternatives (mandibular advancement devices and myofunctional therapy) to the positive airway pressure (PAP) treatment [31]. Unfortunately, the follow-up AHI values were missing; instead, success was measured by “recurrence-free survival”, which refers to the time until either clinical sleep-related symptoms return or AHI increases, as shown by another polysomnogram. The percentage of patients who were objectively (control PSG) rather than subjectively (clinical absence of recurrence) diagnosed with recurrent OSA was therefore unclear. Treatment success rate (defined as a reduction in AHI > 50% from the initial AHI value) was 85% in RME vs. 71.2% in PAP. The follow-up interval was 12 months after RME, immediate after mandibular advancement, but undisclosed for PAP. Longer follow-up times may allow for greater effect of spontaneous OSA improvement [32]. Likewise, a more thorough follow-up in PAP patients may expose them to a higher possibility of being identified as recurrent as compared to orthodontic counterparts that are more likely to be discharged without further control. The use of PAP therapy for mild to moderate pediatric OSA is typically reserved for children with symptoms or comorbidities [33]. Consequently, given the retrospective, non-randomized nature of the study, there may be a selection bias toward patients with a poorer prognosis. Overall, the limitations recognized in these studies exemplify and reinforce the decision not to include retrospective studies in our inclusion criteria for the systematic review. Retrospective designs are inherently unable to disentangle treatment effects from selection bias in a condition as heterogeneous and prone to spontaneous improvement as pediatric OSA. Their exclusion from this review is therefore not merely procedural but reflects a fundamental epistemological requirement: without prospective allocation, any apparent benefit of RME remains confounded.

Maxillary transverse deficiencies, which appear as a narrow or constricted upper jaw or a posterior dental crossbite, are commonly considered to be indicators for RME treatment [34]. These criteria may be subjective and often underreported [12]. In the present review update, two studies reported objective transversal measures of the maxilla; Gokce et al. patients featured maxillary intermolar widths of 42–44 mm and a net width gain of 3 to 5 mm [20]. These data depict a mildly constricted maxilla when compared to normative interfossae molar distances for 12-year-old Caucasians of 45.9 ± 3.7 mm [35]. Aksilp et al. reported maxillary intercanine widths of 32–33 mm and a 4 mm enlargement in the RME group [22], comparable to the maxillary intercanine norm for Asian 6-year-old patients of 32.11 ± 1.95 [36]. Therefore, the previously noted lack of dentofacial criteria disclosure seems to be improving [12], while it continues to represent not particularly transversally constricted maxillae. Notably, in the aforementioned excluded retrospective study by Xia et al., not only did they omit transversal measurements, but they exclusively considered sagittal (anteroposterior) criteria to select treatment candidates. Baseline craniofacial and anthropometric features were not statistically different among all four treatment arms [31]. The lack of description of paramount transverse craniofacial features for the indication of RME treatment raises additional concerns regarding both the inclusion criteria and the reproducibility of the results of this retrospective cohort. A similar limitation in the craniofacial selection criteria—only anteroposterior measurements with no regard to transversal measures whatsoever—was identified in another excluded study [37]. It may be hypothesized that patients had their maxillae transversally expanded irrespective of their transversal features. Providing treatment solely based on an OSA diagnosis (for example, using RME without evidence of transverse discrepancy) is discouraged by recent guidelines [38]. The data from the present update reinforce the concern raised in our original review [12]: patients recruited for RME trials that disclose maxillary dimensions continue to present values within or near normal ranges, which calls into question whether maxillary constriction is the pathophysiological driver of their OSA or merely a co-occurring trait used to justify intervention.

A recently published RCT compared 6- to 8-year-old patients undergoing RME with RME plus a device used to improve tongue position [39]. RME was performed in both arms. Therefore, the difference in treatment efficacy could be only attributed to the myofunctional intervention, while the weight of RME itself in the overall AHI improvement remains as elusive as in uncontrolled case-series [12]. Notably, other RCTs have not found advantages of supervised myofunctional therapy in adult OSA patients [40].

The main limitation of this review, inherited from our original design, is the fact that we relied exclusively on objective sleep study outcomes (AHI, LSAT, and MSAT) to assess intervention efficacy. While AHI, our main outcome of choice, has limitations for establishing both diagnosis and treatment success [41], it remains the most accepted and used measure in pediatric OSA [3]. Most of the evidence ever used to support RME as a treatment for pediatric OSA has relied on the AHI as the main outcome indeed. Therefore, the same level of caution used to evaluate its validity should be exercised when supporting its indication. The controversy on the complexity of OSA diagnosis, its effects over children’s health and quality of life, and its correlation with diverse treatment success thresholds is beyond the scope of this review. There are dozens of screening questionnaires and tools that have been proposed for either surrogate OSA diagnosis or OSA-related symptom relief [12]. Given the significant heterogeneity and the scarcity of appropriate validation of such tools, we opted to stick to objective, widely accepted diagnostic and treatment success criteria. For instance, one study reported subjective outcomes: sleep-related quality of life as measured by a validated OSA-18 questionnaire, improved after treatment in patients of the RCT by Aksilp et al., but the resulting score was significantly (*p* < 0.01) better in the adenotonsillectomy arm (38.42 ± 10.6) than in the RME arm (62.42 ± 9.99), indeed [22]. Additionally, the reporting of objective outcomes is not free of limitations. There is no consensus as to which should be the AHI threshold to diagnose OSA or define treatment success: we have chosen to adopt a widely recognized AHI > 1 [4,14,42], but other thresholds (AHI > 2, AHI > 3, AHI > 5, or even those using obstructive AHI) have been proposed. Furthermore, AHI changes or cure rates were not always appropriately disclosed in the selected studies. When change in AHI was not directly reported, we opted to calculate it using group-level baseline and post-treatment means or medians. This approach may introduce inaccuracy, as percentage change derived from aggregated summary statistics does not reflect individual-level change and does not account for variance, potentially biasing effect estimates. Therefore, we encourage the reader to take this into consideration when analyzing AHI enhancement data or cure rates. Notwithstanding these limitations, the consistency of our findings across two independent systematic review updates—and the fact that the only RCT with a watchful waiting arm [24] found no difference—provides a coherent and reproducible signal: the available evidence does not support RME as an efficacious treatment for pediatric OSA.

By updating our previous systematic review, we have been able to identify new published studies that met our original inclusion criteria; however, comparisons with watchful waiting or supportive care regimes are still critically lacking. As included in our original search protocol, Cochrane Central Registry was consulted for registered ongoing or unpublished research and identified five new studies: two of them compared different types of devices [43,44], and the former has been already published and discussed hereby [39]. The other three studies compared RME with adenotonsillectomy: a parallel RCT [45], crossover RCT [46], and retrospective cohort comparison [47]. None of them featured a watchful waiting or supportive care arm either, thus precluding a true measure of the actual effect of RME in growing pediatric OSA patients. Therefore, the core question remains unanswered: does RME truly add value to the pediatric OSA treatment algorithm? For what patients? Until RCTs with watchful waiting arms are completed and published, any recommendation in favor of RME for pediatric OSA must be considered expert opinion unsupported by controlled evidence—regardless of how many uncontrolled or retrospective studies accumulate in the interim.

## 5. Conclusions

In summary, in this updated systematic review, we were unable to identify convincing evidence of meaningful improvement associated with RME treatment in controlled prospective studies. Comparisons with watchful waiting or supportive care strategies—essential for distinguishing genuine treatment effects from the known potential for spontaneous improvement in pediatric OSA—remain critically scarce. Comparative studies evaluating RME against other treatments have yielded conflicting results, largely due to suboptimal study designs and substantial methodological heterogeneity. Future guidelines and recommendations should emphasize the need for appropriate comparators in randomized research to address this lack of clinically significant evidence of efficacy. In the meantime, caution is warranted when attributing improvements in pediatric OSA to RME treatment.

## Data Availability

The data sources presented in the study are publicly available in the referred databases.

## Supplementary Materials

The following supporting information can be downloaded at: https://www.mdpi.com/article/10.3390/jcm15082976/s1, Supplementary Table S1: PRISMA 2020 checklist; Supplementary Table S2: Search strategy of all databases. Supplementary Table S3: Data items extracted. Supplementary Table S4: Potentially relevant excluded studies [28,29,30,31,33,39,48,49,50,51,52,53,54,55,56,57,58,59,60,61,62,63,64,65,66,67,68,69,70,71,72,73,74,75,76,77,78,79,80,81,82,83,84,85,86,87,88,89,90,91,92,93,94,95,96,97,98]; Supplementary Table S5. Details of rapid maxillary expansion treatment; Supplementary Table S6: JBI critical appraisal checklist for randomized clinical trials; Supplementary Table S7: GRADE staging.

## Abbreviations

The following abbreviations are used in this manuscript:

BMI	body mass index
HSAT	home sleep apnea test
JBI	Joanna Briggs Institute
OSA	obstructive sleep apnea
MSAT	mean oxygen saturation
LSAT	lowest oxygen saturation
PSG	polysomnography
RCT	randomized controlled trial
RME	rapid maxillary expansion
SRDB	sleep-related disordered breathing
T_0_	pre-treatment assessment
T_1_	post-treatment assessment
T&A	adenotonsillectomy
