# Supplementary material for: Controlled Prospective Evidence of Rapid Maxillary Expansion Efficacy in Pediatric Obstructive Sleep Apnea: A Systematic Review Update
Source: J Clin Med. 2026 Apr 14;15(8):2976. doi: 10.3390/jcm15082976 (PMC13116054; doi:10.3390/jcm15082976)
Supplement: Supplementary file 1 [file jcm-15-02976-s001.zip › Supplementary Table S7 v8.pdf]

Journal of Clinical Medicine

MDPI

Supplementary Table S7: GRADE Summary of Findings. **S7.a)** RME versus Active Treatments (T&A, PAP), **S7.b)** RME versus Watchful Waiting (No Direct Evidence Identified)

S7.a) RME versus Active Treatments (T&A, PAP)

Population: Children (<18 years) with OSA diagnosed by PSG or HSAT
Intervention: Rapid Maxillary Expansion (RME)
Comparator: Active treatment (adenotonsillectomy [T&A], positive airway pressure [PAP])
Follow-up: 3–12 months (median longer in retrospective cohort)

Outcomes (Critical/ Important)	No. of Studies (Design)	Participants (n)	Summary of Findings	Certainty Assessment (GRADE Domains)	Overall Certainty
**Change in AHI (critical)**	3 RCTs, 1 retrospective cohort	1319 (90 randomized)	RCTs showed marginal/moderate AHI reduction after RME. T&A demonstrated greater improvement in direct comparisons. Observational data suggested larger effects but with high confounding risk. No pooled estimate due to heterogeneity.	Risk of bias: serious ↓↓; Inconsistency: serious ↓; Indirectness: not serious; Imprecision: serious ↓↓; Publication bias: suspected	⨁◯◯◯ Very low
**Cure rate (AHI <1) (critical)**	2 RCTs	<100	Cure rate numerically higher in T&A compared to RME. No robust statistical superiority demonstrated for RME.	Risk of bias: serious ↓↓; Inconsistency: not serious; Indirectness: not serious; Imprecision: serious ↓↓; Publication bias: undetected	⨁◯◯◯ Very low
**Lowest oxygen saturation (LSAT) (important)**	3 studies	Variable	Minimal or non-clinically meaningful improvements after RME. T&A showed greater improvements in available comparisons.	Risk of bias: serious ↓↓; Inconsistency: serious ↓; Indirectness: not serious; Imprecision: serious ↓; Publication bias: unclear	⨁◯◯◯ Very low

**Mean oxygen saturation (MSAT) (important)**	2 studies	Variable	No clinically relevant differences between RME and comparators.	Risk of bias: serious ↓↓; Inconsistency: not serious; Indirectness: not serious; Imprecision: serious ↓; Publication bias: unclear	⊕◯◯◯ Very low

S7.b) RME versus Watchful Waiting (No Direct Evidence Identified)

Population: Children (<18 years) with OSA diagnosed by PSG or HSAT
Intervention: Rapid Maxillary Expansion (RME)
Comparator: Watchful waiting / supportive care
Search updated until February 2026
Result: No eligible controlled trials comparing RME with watchful waiting were identified.

Outcomes (Critical/ Important)	No. of Studies (Design)	Participants (n)	Summary of Findings	Certainty Assessment (GRADE Domains)	Overall Certainty
**Change in AHI (critical)**	0	—	No direct evidence available.	No direct comparisons identified. Certainty cannot be estimated.	⊕◯◯◯ Very low*
**Cure rate (AHI <1) (critical)**	0	—	No direct evidence available.	No direct comparisons identified. Certainty cannot be estimated.	⊕◯◯◯ Very low*
**Lowest oxygen saturation (LSAT) (important)**	0	—	No direct evidence available.	No direct comparisons identified. Certainty cannot be estimated.	⊕◯◯◯ Very low*
**Mean oxygen saturation (MSAT) (important)**	0	—	No direct evidence available.	No direct comparisons identified. Certainty cannot be estimated.	⊕◯◯◯ Very low*

Abbreviations: AHI, apnea–hypopnea index; RME, rapid maxillary expansion; T&A, adenotonsillectomy; PAP, positive airway pressure; RCT, randomized controlled trial.
