# Supplementary material for: Controlled Prospective Evidence of Rapid Maxillary Expansion Efficacy in Pediatric Obstructive Sleep Apnea: A Systematic Review Update
Source: J Clin Med. 2026 Apr 14;15(8):2976. doi: 10.3390/jcm15082976 (PMC13116054; doi:10.3390/jcm15082976)
Supplement: Supplementary file 1 [file jcm-15-02976-s001.zip › Supplementary Table S2 v7.pdf]

Journal of Clinical Medicine

MDPI

Supplementary table S2. Search strategy of all databases.

Databases	Search strategy
World of Science (WOS) Indexes: *SCI-EXPANDED, SSCI, A&HCI, CPCI-S, CPCI-SSH, BKCI-S, BKCI-SSH, ESCI, CCR-EXPANDED*	#1: (maxil* OR palat*)<br>#2: (expansion OR rme OR rpe)<br>#3: (OSA* OR Apnea OR sleep OR SRBD OR SDB* OR Sleep Related Breathing Disorders)<br>#4: #3 AND #2 AND #1<br>Timespan: December 1, 2021 – February 1, 2026
OVID Indexes: *EMBASE and Medline/Pubmed*	#1: (palat* or maxil*).mp.<br>#2: (expansion or RME or RPE).mp.<br>#3: (OSA* or sleep or apnea or SDB or SRBD or Sleep related breathing disorders).mp.<br>#4: #1 AND #2 AND #3<br>#5: limit #4 to yr="2021 -Jan 2026"
COCHRANE CENTRAL	#1: palat* OR maxil*<br>#2: expansion OR RME OR RPE<br>#3: OSA* OR sleep OR apnea OR SDB OR SRBD OR Sleep related breathing disorders<br>#4: #1 AND #2 AND #3<br>Limit: December 2021-January 2026
