# Supplementary material for: Controlled Prospective Evidence of Rapid Maxillary Expansion Efficacy in Pediatric Obstructive Sleep Apnea: A Systematic Review Update
Source: J Clin Med. 2026 Apr 14;15(8):2976. doi: 10.3390/jcm15082976 (PMC13116054; doi:10.3390/jcm15082976)
Supplement: Supplementary file 1 [file jcm-15-02976-s001.zip › Supplementary Table S3 v8.pdf]

Journal of Clinical Medicine

Supplementary table S3. Data items extracted.

Fields	Data extracted
Demographic	Patients screened/recruited, recruiting period, age, sex of patients, lost to follow-up
Anthropometric	BMI
Craniofacial	Posterior cross-bite, narrow palate, maxillary intercanine width, maxillary intermolar width, ojival palate, deep bite, overbite, maxillary hypoplasia, mandibular hypoplasia, overjet, angle class
Treatment Technique	Type of device, type of bonding, activation schedule, activation period, maxillary intercanine and intermolar width gain, retention period, expansion maintained, adverse outcome register, adverse outcomes, alternative treatment delivered.
Diagnosis	Type of sleep study (PSG/HSAT), scoring criteria, pre and post-treatment sleep parameters and changes in intervention and control arms (AHI, obstructive AHI, oxygen desaturation index, lowest oxygen saturation, mean oxygen saturation), residual disease after treatment, percentage of change in AHI before and after treatment, time interval between initial and final sleep study.

AHI: Apnea hypopnea index; BMI: Body mass index; HSAT: Home sleep apnea test; PSG: Polysomnography.

 https://doi.org/10.3390/xxxxx
