# Supplementary material for: Controlled Prospective Evidence of Rapid Maxillary Expansion Efficacy in Pediatric Obstructive Sleep Apnea: A Systematic Review Update
Source: J Clin Med. 2026 Apr 14;15(8):2976. doi: 10.3390/jcm15082976 (PMC13116054; doi:10.3390/jcm15082976)
Supplement: Supplementary file 1 [file jcm-15-02976-s001.zip › Supplementary Table S4 v8.pdf]

Journal of
Clinical Medicine

MDPI

Supplementary table S4. Potentially relevant excluded studies.

Reasons for exclusion	Excluded studies
Uncontrolled	Kim *et al.* [48], Remy *et al.* [49], Pirelli *et al.* [50], Kushida *et al.* [51], Li *et al.* [52], Pirelli *et al.* [53], Bariani *et al.* [54], Bittencourt *et al.* [55], Meira *et al.* [56], Mastud *et al.* [57], Yang *et al.* [33], Bavani *et al.* [58], Restrepo *et al.* [39]. Bellerive et al.[59], Fastuca *et al.* [60], Fastuca *et al.* [61], Caprioglio *et al.* [62], Schutz *et al.* [63]; Buccheri *et al.* [64], Pirelli *et al.*[65], Villa *et al.*[66], Guilleminault *et al.*[67], Marino *et al.*[68], Villa *et al.* [69], Villa *et al.* [70], Pirelli *et al.* [71], Pirelli *et al.* [72]
No sleep study or no OSA diagnostic criteria	Remy *et al.* [28], Alforaidi *et al.* [29], Yoon *et al.* [30], Galeotti *et al.* [73], Shetty *et al.* [74], Zhao et al. [76], Miano et al. [77], Zhang et al. [78], роедова et al. [79], Izuka et al. [80]
Case reports	Alexander *et al.* [81], Bonetti *et al.* [82], Rose *et al.* [83], Li *et al.* [84], Gracco *et al.* [85], Galeotti *et al.* [86]
Syndromes	Peanchitlertkajorn *et al.* [87], de Moura et al. [88], Taddei *et al.* [89]
Adults	Dao *et al.* [90]
Retrospective	Xia *et al.* [31]
Study protocols	Gökçe *et al.* [91], Valladares Neto [92], Gökçe *et al.*[93], Pliska et al. [94], Cunha *et al.* [95], Monteiro *et al.*[96], Fernández-Barriales *et al.* [97], Machado Junior *et al.* [98], Capenakas *et al.* 2019 [99], Liu *et al.* [100].

28. Remy F, Boyer E, Daniel C, Rousval E, Moisdon P, Burgart P, Bonnaure P, Godio-Raboutet Y, Guyot L, Meuric V, Thollon L. Management of the pediatric OSAS: what about simultaneously expand the maxilla and advance the mandible? A retrospective non-randomized controlled cohort study. *Sleep Med* **2022**,90,135-141. doi: 10.1016/j.sleep.2022.01.007.
29. Alforaidi S, Zreaqat M, Hassan R. Effects of Rapid Maxillary Expansion on Urinary Leukotriene E4 and Serum C-Reactive Protein Levels in Children With Obstructive Sleep Apnea and Maxillary Restriction: A Prospective Longitudinal Study. *Pediatr Pulmonol* **2025**,60(8),e71235. doi: 10.1002/ppul.71235.
30. Yoon A, Abdelwahab M, Bockow R, Vakili A, Lovell K, Chang I, Ganguly R, Liu SY, Kushida C, Hong C. Impact of rapid palatal expansion on the size of adenoids and tonsils in children. *Sleep Med* **2022**,92,96-102. doi: 10.1016/j.sleep.2022.02.011.
31. Xia T, Luo R, Wan M. Long-term recurrence-free survival of orthodontic treatments for Class II malocclusion as the important supplement protocol to adenotonsillectomy in children with obstructive sleep apnea syndrome: a case-control retrospective study. *J Clin Pediatr Dent* **2025**,49 (2),178-187. doi: 10.22514/jocpd.2025.037.
48. Kim JE, Hwang KJ, Kim SW, et al. Correlation between craniofacial changes and respiratory improvement after nasomaxillary skeletal expansion in pediatric obstructive sleep apnea patients. *Sleep Breath* **2022**;26:585–594. https://doi.org/10.1007/s11325-021-02426-9.
49. Remy F, Bonnaure P, Moisdon P, Burgart P, Godio-Raboutet Y, Thollon L, Guyot L. Preliminary results on the impact of simultaneous palatal expansion and mandibular advancement on the respiratory status recorded during sleep in OSAS children. *J Stomatol Oral Maxillofac Surg* **2021**;122(3):235-240. doi: 10.1016/j.jormas.2020.07.008.

50. Pirelli P, Fiaschetti V, Fanucci E, Giancotti A, Condo' R, Saccomanno S, Mampieri G. Cone beam CT evaluation of skeletal and nasomaxillary complex volume changes after rapid maxillary expansion in OSA children. *Sleep Med* **2021**;86:81-89. doi: 10.1016/j.sleep.2021.08.011.
51. Kushida CA, Stevens J, Bennett M, Heit T, Klemp D, Raio D, Cozean J, Cozean C. Multicenter clinical trial for the treatment of obstructive sleep apnea with a non-permanent orthodontic intraoral device in children. *Eur J Pediatr* **2025**;184(7):424. doi: 10.1007/s00431-025-06254-x.
52. Li K, Iwasaki T, Quo S, Li C, Young K, Leary E, Guilleminault C. Persistent pediatric obstructive sleep apnea treated with skeletally anchored transpalatal distraction. *Orthod Fr* **2022**;93(Suppl 1):47-60. doi: 10.1684/orthodfr.2022.86.
53. Pirelli P, Fiaschetti V, Mampieri G, Condo' R, Ubaldi N, Pachi F, Giancotti A. Effect of rapid maxillary expansion on naso-maxillary structure and sleep disordered breathing in children with obstructive sleep apnoea. *Aust Dent J* **2024**;69 Suppl 1:S112-S120. doi: 10.1111/adj.13049.
54. Bariani RCB, Bigliazzi R, Badreddine FR, Yamamoto LH, Tufik S, Moreira G, Fujita RR. A clinical trial on 3D CT scan and polysomnographyc changes after rapid maxillary expansion in children with snoring. *Braz J Otorhinolaryngol* **2022**;88 Suppl 5(Suppl 5):S162-S170. doi: 10.1016/j.bjorl.2022.04.004.
55. Bittencourt ABBC, Melo-Neto CLM, Dos Santos GA, da Silva EVF, Muraoka CSAS, Bertoz APM, Dos Santos DM, Goiato MC. Myofunctional Responses in Obstructive Sleep Apnea Syndrome in Children Following the Use of Two Oral Orthopedic Devices. *J Clin Exp Dent 2025*;17(4):e393-e398. doi: 10.4317/jced.62603.
56. Meira E Cruz M, Guilleminault C, Gozal D, Bruni O, Pirelli P. Rapid maxillary expansion in COMISA: preliminary observations from a 12 years follow-up. *Sleep* **2025**;48(3):zsae306. doi: 10.1093/sleep/zsae306.
57. Mastud CS, Deshmukh SV, Rahalkar J, Bharatwal M, Mane S, Mastud SP. Evaluation of treatment outcomes of customized fixed intra-oral appliance with maxillary expansion and twin block in pediatric obstructive sleep apnea patients: A prospective study. *Sleep Med Res* **2024**;15(2):113-123. doi: 10.17241/smr.2024.02124.
58. Bavani SZ, Ng ET, Vich MOL. Rapid maxillary expansion and its impact on sleep apnea in children aged 5 to 8 years: a retrospective study. *Dental Press J Orthod* **2025**;30(5):e2524280. doi: 10.1590/2177-6709.30.5.e2524280.oar.
59. Bellerive A, Montpetit A, El-Khatib H, Carra MC, Remise C, Desplats E, et al. The effect of rapid palatal expansion on sleep bruxism in children. *Sleep Breath* **2015**;19(4):1265-71. doi: 10.1007/s11325-015-1156-4.
60. Fastuca R, Perinetti G, Zecca PA, Nucera R, Caprioglio A. Airway compartments volume and oxygen saturation changes after rapid maxillary expansion: a longitudinal correlation study. *Angle Orthod* **2015**;85(6):955-61. doi: 10.2319/072014-504.1.
61. Fastuca R, Meneghel M, Zecca PA, Mangano F, Antonello M, Nucera R, et al. Multimodal airway evaluation in growing patients after rapid maxillary expansion. *Eur J Paediatr Dent* **2015**;16(2):129-34.
62. Caprioglio A, Meneghel M, Fastuca R, Zecca PA, Nucera R, Nosetti L. Rapid maxillary expansion in growing patients: correspondence between 3-dimensional airway changes and polysomnography. *Int J Pediatr Otorhinolaryngol* **2014**;78(1):23-7. doi: 10.1016/j.ijporl.2013.10.011.
63. Schütz TC, Dominguez GC, Hallinan MP, Cunha TC, Tufik S. Class II correction improves nocturnal breathing in adolescents. *Angle Orthod* **2011**;81(2):222-8. doi: 10.2319/052710-233.1.
64. Buccheri A, Chinè F, Fratto G, Manzon L. Rapid Maxillary Expansion in Obstructive Sleep Apnea in Young Patients: Cardio-Respiratory Monitoring. *J Clin Pediatr Dent* **2017**;41(4):312-316. doi: 10.17796/1053-4628-41.4.312
65. Pirelli P, Saponara M, Guilleminault C. Rapid maxillary expansion (RME) for pediatric obstructive sleep apnea: a 12-year follow-up. *Sleep Med* **2015**;16(8):933-5. doi: 10.1016/j.sleep.2015.04.012. Epub 2015 May 19.
66. Villa MP, Rizzoli A, Rabasco J, Vitelli O, Pietropaoli N, Cecili M, et al. Rapid maxillary expansion outcomes in treatment of obstructive sleep apnea in children. *Sleep Med* **2015**;16(6):709-16. doi: 10.1016/j.sleep.2014.11.019
67. Guilleminault C, Huang YS, Quo S, Monteyrol PJ, Lin CH. Teenage sleep-disordered breathing: recurrence of syndrome. *Sleep Med* **2013**;14(1):37-44. doi: 10.1016/j.sleep.2012.08.010. Epub 2012 Sep 29. Erratum in: Sleep Med. 2013 Sep;14(9):927-8.
68. Marino A, Ranieri R, Chiarotti F, Villa MP, Malagola C. Rapid maxillary expansion in children with Obstructive Sleep Apnoea Syndrome (OSAS). *Eur J Paediatr Dent* **2012**;13(1):57-63.
69. Villa MP, Rizzoli A, Miano S, Malagola C. Efficacy of rapid maxillary expansion in children with obstructive sleep apnea syndrome: 36 months of follow-up. *Sleep Breath* **2011**;15(2):179-84. doi: 10.1007/s11325-011-0505-1

70. Villa MP, Malagola C, Pagani J, Montesano M, Rizzoli A, Guilleminault C, et al. Rapid maxillary expansion in children with obstructive sleep apnea syndrome: 12-month follow-up. *Sleep Med* **2007**;8(2):128-34. doi: 10.1016/j.sleep.2006.06.009
71. Pirelli P, Saponara M, Guilleminault C. Rapid maxillary expansion in children with obstructive sleep apnea syndrome. *Sleep* **2004**;27(4):761-6. doi: 10.1093/sleep/27.4.761
72. Pirelli P, Saponara M, De Rosa C, Fanucci E. Orthodontics and obstructive sleep apnea in children. *Med Clin North Am* **2010**;94(3):517-29. doi: 10.1016/j.mcna.2010.02.004.
73. Galeotti A, Gatto R, Caruso S, Piga S, Maldonato W, Sitzia E, Viarani V, Bompiani G, Aristei F, Marzo G, Festa P. Effects of Rapid Palatal Expansion on the Upper Airway Space in Children with Obstructive Sleep Apnea (OSA): A Case-Control Study. *Children (Basel)* **2023**,10(2),244. doi: 10.3390/children10020244.
74. Shetty A, Ratti S, Nakra P, Shetty S, Mohammed A, Saidath K. Evaluation of Soft Tissue and Airway Changes in Individuals Treated with Mini-Implant Assisted Rapid Palatal Expansion (MARPE). *J Long Term Eff Med Implants* **2022**;32(1):7-18. doi: 10.1615/JLongTermEffMedImplants.2021038874.
75. Zhao T, Ngan P, Hua F, Zheng J, Zhou S, Zhang M, et al. Impact of pediatric obstructive sleep apnea on the development of Class II hyperdivergent patients receiving orthodontic treatment: A pilot study. *Angle Orthod* **2018**;88(5):560-566. doi: 10.2319/110617-759.1
76. Miano S, Rizzoli A, Evangelisti M, Bruni O, Ferri R, Pagani J, et al. NREM sleep instability changes following rapid maxillary expansion in children with obstructive apnea sleep syndrome. *Sleep Med* **2009**;10(4):471-8. doi: 10.1016/j.sleep.2008.04.003
77. Zhang X, He JM, Zheng WY. Comparison of rapid maxillary expansion and pre-fabricated myofunctional appliance for the management of mouth breathers with Class II malocclusion. *Eur Rev Med Pharmacol Sci* **2021**;25(1):16-23. doi: 10.26355/eurrev_202101_24340
78. Куроедова ВД, Чикор ТА, Макарова АН, Ким АА. [Orthodontic treatment effect of on the condition of the upper airways]. *Wiad Lek* **2016**;69(6):734-736.
79. Izuka EN, Feres MF, Pignatari SS. Immediate impact of rapid maxillary expansion on upper airway dimensions and on the quality of life of mouth breathers. *Dental Press J Orthod* **2015**;20(3):43-9. doi: 10.1590/2176-9451.20.3.043-049.oar
80. Alexander N, Boota A, Hooks K, White JR. Rapid Maxillary Expansion and Adenotonsillectomy in 9-Year-Old Twins With Pediatric Obstructive Sleep Apnea Syndrome: An Interdisciplinary Effort. *J Am Osteopath Assoc* **2019**;119(2):126-134. doi: 10.7556/jaoa.2019.019
81. Bonetti GA, Piccin O, Lancellotti L, Bianchi A, Marchetti C. A case report on the efficacy of transverse expansion in severe obstructive sleep apnea syndrome. *Sleep Breath* **2009**;13(1):93-6. doi: 10.1007/s11325-008-0206-6
82. Rose E, Schessl J. Orthodontic procedures in the treatment of obstructive sleep apnea in children. *J Orofac Orthop* **2006**;67(1):58-67. English, German. doi: 10.1007/s00056-006-0534-8
83. Li KK, Riley R, Powell N, Hester J. Skeletal expansion by gradual intraoral distraction osteogenesis for the treatment of obstructive sleep apnea. *Operative Techniques in Otolaryngology-Head and Neck Surgery* **2002**;13(2):119-122. doi:10.1053/otot.2002.127288
84. Gracco A, Bruno G, de Stefani A, Ragona RM, Mazzoleni S, Stellini E. Combined Orthodontic and Surgical Treatment in a 8-Years-Old Patient Affected By Severe Obstructive Sleep Apnea: A Case-Report. *J Clin Pediatr Dent* **2018**;42(1):79-84. doi: 10.17796/1053-4628-42.1.14
85. Galeotti A, Festa P, Pavone M, De Vincentiis GC. Effects of simultaneous palatal expansion and mandibular advancement in a child suffering from OSA. *Acta Otorhinolaryngol Ital* **2016**;36(4):328-332. doi: 10.14639/0392-100X-548
86. Peanchitlertkajorn S, Assawakawintip T, Pibulniyom M, Srisan P, Pungchanchaikul P, Jaroenying R. Successful treatment of a child with Schwartz-Jampel syndrome using rapid maxillary expansion and CPAP. *J Clin Sleep Med* **2021**;17(3):601-604. doi: 10.5664/jcsm.9028
87. de Moura CP, Andrade D, Cunha LM, Tavares MJ, Cunha MJ, Vaz P, et al. Down syndrome: otolaryngological effects of rapid maxillary expansion. *J Laryngol Otol* **2008**;122(12):1318-24. doi: 10.1017/S002221510800279X.

88. Taddei M, Alkhamis N, Tagariello T, D'Alessandro G, Mariucci EM, Piana G. Effects of rapid maxillary expansion and mandibular advancement on upper airways in Marfan's syndrome children: a home sleep study and cephalometric evaluation. *Sleep Breath* **2015**;19(4):1213-20. doi: 10.1007/s11325-015-1141-y
89. Dao N, Cozean C, Chernyshev O, Kushida C, Greenburg J, Alexander JS. Retrospective Analysis of Real-World Data for the Treatment of Obstructive Sleep Apnea with Slow Maxillary Expansion Using a Unique Expansion Dental Appliance (DNA). *Pathophysiology* **2023**;30(2):199-208. doi: 10.3390/pathophysiology30020017.
90. Gökçe G. Evaluation of the effects of different rapid maxillary expansion appliances on obstructive sleep apnea. 2020 Oct 27 [last updated 2026 Feb 1; cited 2026 Mar 11]. In: ClinicalTrials.gov [Internet]. Bethesda (MD): U.S. National Library of Medicine. Available from: https://clinicaltrials.gov/ct2/show/NCT04604392 Identifier: NCT04604392.
91. Valladares Neto J. Maxillary expansion effects in children with upper airway obstruction. 2016 Dec 28 [last updated 2026 Feb 1; cited 2026 Mar 11]. In: ClinicalTrials.gov [Internet]. Bethesda (MD): U.S. National Library of Medicine. Available from: https://clinicaltrials.gov/ct2/show/NCT03004300 Identifier: NCT03004300.
92. Gökçe G. Polygraphic evaluation of the effects of different rapid maxillary expansion appliances on sleep quality. 2020 Aug 27 [last updated 2026 Feb 1; cited 2026 Mar 11]. In: ClinicalTrials.gov [Internet]. Bethesda (MD): U.S. National Library of Medicine. Available from: https://clinicaltrials.gov/ct2/show/NCT04529213 Identifier: NCT04529213.
93. Pliska B. Maxillary Expansion Treatment of Pediatric OSA. 2013 April 23 [last updated 2026 Feb 1; cited 2026 Mar 11]. In: ClinicalTrials.gov [Internet]. Bethesda (MD): U.S. National Library of Medicine. Available from: https://clinicaltrials.gov/ct2/show/NCT01837914 Identifier: NCT01837914.
94. Cunha TCA, Almeida GR, Novaes RM, Backin F, Magalhaes MCM, Lopes AJ et al. Treatment of Childhood Obstructive Sleep Apnea - Adenotonsilectomy X Rapid Maxillary Expansion - Prospective, randomized, crossover study - Partial Results. Sleep Sci. 2019;12(Supl.1):1-82. https://cdn.publisher.gn1.link/sleepscience.org.br/pdf/v12s1a02.pdf
95. Monteiro MC. Impact of tonsil and adenoid removal surgery and maxillary expansion on respiratory capacity in children with Obstructive Sleep Apnea Syndrome (OSA) - randomized clinical study. 2020 Sep 29 [last updated 2020 Sep 29; cited 2021 Dec 1]. In: ensaiosclinicos.gov.br [Internet]. Available from: https://ensaiosclinicos.gov.br/rg/RBR-5wq5s9 Identifier: RBR-5wq5s9.
96. Fernández-Barriales M. Rapid Maxillary Expansion for Residual Pediatric (ERMES). 2016 Aug 28 [last updated 2026 Feb 1; cited 2026 Mar 11]. In: ClinicalTrials.gov [Internet]. Bethesda (MD): U.S. National Library of Medicine. Available from: https://clinicaltrials.gov/ct2/show/NCT02947464 Identifier: NCT02947464.
97. Machado Junior AJ. Study in children with Obstructive Sleep Apnea, after surgery, treated with a dental appliance or speech therapy. 2020 Mar 20 [last updated 2026 Feb 1; cited 2026 Mar 11]. In: ensaiosclinicos.gov.br [Internet]. Available from: https://ensaiosclinicos.gov.br/rg/RBR-222dr8 Identifier: RBR-222dr8.
98. Capenakas SG. Upper Airway's Pressure Drop Analyses After Mandibular Advancement and Maxillary Expansion. 2019 Dec 9 [last updated 2026 Feb 1; cited 2026 Mar 111]. In: ClinicalTrials.gov [Internet]. Bethesda (MD): U.S. National Library of Medicine. Available from: https://clinicaltrials.gov/ct2/show/NCT04190953 Identifier: NCT04190953.
99. Liu Y. Multi-disciplinary Diagnosis and Treatment Process and Evaluation System for Children With Sleep Disordered Breathing and Malocclusion. 2018 March 1 [last updated 2026 Feb 1; cited 2026 Mar 11]. In: ClinicalTrials.gov [Internet]. Bethesda (MD): U.S. National Library of Medicine. Available from: https://clinicaltrials.gov/ct2/show/NCT03451318 Identifier: NCT03451318.
