# Supplementary material for: Controlled Prospective Evidence of Rapid Maxillary Expansion Efficacy in Pediatric Obstructive Sleep Apnea: A Systematic Review Update
Source: J Clin Med. 2026 Apr 14;15(8):2976. doi: 10.3390/jcm15082976 (PMC13116054; doi:10.3390/jcm15082976)
Supplement: Supplementary file 1 [file jcm-15-02976-s001.zip › Supplementary Table S5 v8.pdf]

Supplementary Table S5. Details of rapid maxillary expansion treatment.

	Dentofacial criteria	Intraoral device	Activation	Retention
Guilleminault et al. 2011 [23]	Narrow maxilla associated with a high and narrow hard palate, as determined by an orthodontist	Appliance designed to be fixed to the teeth.	Expansion rates were typically 0.25mm per day, as measured at the appliance	Not reported
Hoxha et al. 2018 [24]	Clinical signs of maxillary transverse deficiency, malocclusion (high, narrow palate associated with deep bite, retrusive bite, or cross-bite)	A modified McNamara RME device was cemented with an expansion screw connecting right and left maxillary dental acrylic segments to each other with a Hyrax type maxi screw (Forestadent, Pforzheim, Germany)	The screw was turned twice a day for the first 7 days, then once a day until the palatal cusp of the upper molar came into contact with the buccal cusp of the lower molar	5.01 ± 0.96 months
Pirelli et al. 2012 [25]	Narrow upper jaw at examination, diagnosed clinically and confirmed by cephalometric assessment according to Ricketts parameters	Fixed two-band appliance with an expansion screw anchored on selected teeth (first molars and permanent premolars in mixed dention; second primary molars in deciduous dentition)	Day 1 (morning and evening) three consecutive activations at 10 min intervals, and from day 2 onward, one activation every morning and evening (1 turn = 0,25mm)	At least 3-4 months
Villa et al. 2014 [26]	High-arched palate and/or malocclusions, and dysgnathia, according to the orthodontist's evaluation	Fixed two-band RME appliance (Leone Sesto Fiorentino, Florence, Italy) with an expansion screw fitted to the second deciduous molars of the upper jaw.	The screw was turned two turns a day for the first 10 days until the palatal cusp of the upper molar came into contact with the buccal cusp of the lower molar.	12 months
Villa et al. 2016 [27]	Dental malocclusion	Fixed two-band RME appliance (Leone Sesto Fiorentino, Florence, Italy) with an expansion screw fitted to the second deciduous molars of the upper jaw.	The screw was turned two turns a day for the first 10 days until the palatal cusp of the upper molar came into contact with the buccal cusp of the lower molar.	Not reported
Gokce et al. [20]	Bilateral crossbite and necessity for rapid maxillary expansion	Nine-millimeter Hyrax expansion screw ((G&H Orthodontics, Franklin, IN, USA)<br>TTB: the occlusal and half of the palatinal and buccal faces of the molar and premolar teeth were coated by heat-polymerizing acrylic (Vertex Dental, Soesterberg, The Netherlands), and attached to the posterior teeth using glass ionomer cement (Ketac Cem, 3M ESPE, Seefeld, Germany).<br>TB: long parts of the expansion screw are soldered to molar and premolar teeth bands and attached to	2 turns per day until the palatal tubercles of the upper molars reached contact with the buccal tubercles of the lower molars.	3 months

		posterior teeth using glass ionomer cement (Ketac Cem, 3 M ESPE, Seefeld, Germany).<br>BB: two mini-screws 1.6 mm, 10 mm (Tomas, Dentaurum, Ispringen, Germany) were settled with 60–70° angle bilaterally among the roots of the 2nd premolar and 1st molar teeth. Then, heat-polymerizing acrylic (Vertex Dental, Soesterberg, The Netherlands) was used to cover the screws and partially the palatal area. Consequently, the appliance was attached to the mini-screws in the palatinal area using the light-cured composite (Transbond XT, 3M Unitek, Monrovia, CA, USA).		
Magalhaes et al. [21]	Constricted maxillary arch, high palate, with a unilateral or bilateral posterior crossbite, determined by the anatomical morphological clinical evaluation of the maxilla (presence of triangular morphology of dental arch and palate, excessive lingual inclination of upper posterior teeth, and/or presence of a buccal corridor during smile), and a reasonable maxillomandibular sagittal and vertical relationship, established by the frontal (the balance between the upper, middle, and lower thirds of the face) and profile facial analysis and confirmed by the assessment of the maxillomandibular relationship cephalometric measurements (SNA, SNB, ANB, and Wits).	Conventional Hyrax maxillary expander composed of an 11-mm screw centrally positioned on the palate.	2 turns per day until obtaining an overcorrection of approximately 2 to 3mm at the level of the permanent molars.	6 months
Aksilp et al. [22]	Narrow and constricted maxilla with or without posterior dental crossbite according to an orthodontic evaluation.	Mid-palatal expansion screw Hyrax, Maxi-12 (Dentaurum, Ispringen, Germany) with arm extensions from anchor teeth to canine, anchored either the upper first permanent molars or the upper second primary molars, depending on the participant’s stage of dental development.	2 turns per day for 14 days (7 mm of expansion).	6 months

TTB tooth-tissue-borne; TB: tooth-borne; BB: Bone-borne. Grey shading indicates studies included in the original review **[12]**
