# Supplementary material for: Controlled Prospective Evidence of Rapid Maxillary Expansion Efficacy in Pediatric Obstructive Sleep Apnea: A Systematic Review Update
Source: J Clin Med. 2026 Apr 14;15(8):2976. doi: 10.3390/jcm15082976 (PMC13116054; doi:10.3390/jcm15082976)
Supplement: Supplementary file 1 [file jcm-15-02976-s001.zip › Supplementary Table S6 v8.pdf]

Journal of
Clinical Medicine

MDPI

Supplementary table S6. JBI critical appraisal checklist for a) randomized clinical trials and b) quasi-experimental designs

a) JBI critical appraisal checklist for **randomized clinical trials**

#		Guilleminault et al. [23]	Hoxha et al. [24]	Gokce et al. [20]	Magalhães et al. [21]	Aksilp et al. [22]
1	Was true randomization used for assignment of participants to treatment groups?	U	NA	Y	Y	Y
2	Was allocation to treatment groups concealed?	U	NA	Y	Y	U
3	Were treatment groups similar at the baseline?	Y	Y	Y	Y	Y
4	Were participants blind to treatment assignment?	N	N	Y	Y	N
5	Were those delivering treatment blind to treatment assignment?	N	N	N	N	N
6	Were outcomes assessors blind to treatment assignment?	U	NA	Y	N	U
7	Were treatment groups treated identically other than the intervention of interest?	NA	NA	Y	Y	Y
8	Was follow up complete and if not, were differences between groups in terms of their follow up adequately described and analyzed?	Y	Y	Y	Y	Y
9	Were participants analyzed in the groups to which they were randomized?	Y	Y	U	Y	U
10	Were outcomes measured in the same way for treatment groups?	Y	Y	Y	U	Y
11	Were outcomes measured in a reliable way?	Y	Y	Y	Y	Y
12	Was appropriate statistical analysis used?	Y	Y	Y	Y	Y
13	Was the trial design appropriate, and any deviations from the standard RCT design (individual randomization, parallel groups) accounted for in the conduct and analysis of the trial?	Y	Y	U	U	Y

N: No; NA: not answered; U: Unclear; Y: Yes.

b) JBI critical appraisal checklist for **quasi-experimental designs**

#		Pirelli et al. [25]	Villa et al. 2014 [26]	Villa et al. 2016 [27]
1	Is it clear in the study what is the 'cause' and what is the 'effect' (i.e. there is no confusion about which variable comes first)?	Y	Y	Y

2	Were the participants included in any comparisons similar?	Y	N	N
3	Were the participants included in any comparisons receiving similar treatment/care, other than the exposure or intervention of interest?	N	U	U
4	Was there a control group?	Y	Y	Y
5	Were there multiple measurements of the outcome both pre and post the intervention/exposure?	Y	N	N
6	Was follow up complete and if not, were differences between groups in terms of their follow up adequately described and analyzed?	Y	Y	Y
7	Were the outcomes of participants included in any comparisons measured in the same way?	Y	Y	Y
8	Were outcomes measured in a reliable way?	U	Y	U
9	Was appropriate statistical analysis used?	Y	Y	Y

N: No; NA: not answered; U: Unclear.; Y: Yes.
